# Glucocorticoid excess induces long-lasting changes in body composition in male C57Bl/6J mice only with high-fat diet

**DOI:** 10.1002/phy2.103

**Published:** 2013-10-11

**Authors:** Hanna E Auvinen, Claudia P Coomans, Mariëtte R Boon, Johannes A Romijn, Nienke R Biermasz, Onno C Meijer, Louis M Havekes, Johannes W A Smit, Patrick C N Rensen, Alberto M Pereira

**Affiliations:** 1 Department of Endocrinology and Metabolic Diseases, Leiden University Medical CenterPO Box 9600, Leiden, 2300 RC, The Netherlands; 2 Department of Molecular Cell Biology, Leiden University Medical CenterPO Box 9600, Leiden, 2300 RC, The Netherlands; 3 Einthoven Laboratory for Experimental Vascular Medicine, Leiden University Medical CenterPO Box 9600, Leiden, 2300 RC, The Netherlands; 4 Department of Medicine, Academic Medical CenterPO Box 22660, Amsterdam, 1100 DD, The Netherlands; 5 TNO-Metabolic Health Research, Gaubius LaboratoryPO Box 2215, Leiden, 2333 CK, The Netherlands; 6 Department of Internal Medicine, Radboud University Medical CenterPO Box 9101, Nijmegen, 6500 HB, The Netherlands

**Keywords:** Body composition, corticosterone, metabolic syndrome, mice

## Abstract

Glucocorticoid (GC) overexposure period as observed in Cushing's syndrome (CS) is associated with the metabolic syndrome and cardiovascular disease, which persist after long-term correction of GC excess. We performed a mouse study to identify factors that modulate metabolic recovery from a GC overexposure period. Male C57Bl/6J mice, fed a low-fat diet (LFD) or a high-fat diet (HFD), received corticosterone (CORT) (50 μg/mL) or vehicle in the drinking water for 4 weeks, followed by an 8-week washout period. Plasma circadian CORT, lipids, insulin, and glucose levels were assessed regularly. Hyperinsulinemic-euglycemic clamp and body composition were analyzed at week 12 under anesthesia. CORT treatment increased plasma CORT levels, food intake, and plasma insulin and lipid levels on both diets. CORT treatment abrogation normalized CORT levels, food intake, and body weight, whereas plasma insulin levels remained significantly higher in CORT-treated mice on both diets. Only on a HFD, CORT-treated mice had decreased lean body mass and higher fat mass. In conclusion, CORT excess period induces long-lasting metabolic changes and some are present only on a HFD. These observations indicate that diet-dependent CORT effects might contribute to the adverse cardiovascular risk profile observed in CS patients, and possibly also in subjects exposed to chronic stress.

## Introduction

In humans, prolonged excessive exposure to glucocorticoids (GC) as seen in Cushing's syndrome (CS) is associated with an increased incidence of the metabolic syndrome (MetS), a clustering of cardiovascular risk factors such as increased blood pressure, dyslipidemia, and insulin resistance, and increased cardiovascular morbidity and mortality (Newell-Price et al. 2006). Increased GC exposure changes eating behavior and food preferences facilitating the development of obesity and the MetS (Dallman et al. 2003; Warne et al. 2009). Intriguingly, patients with CS remain at increased cardiovascular risk, even despite long-term successful correction of GC excess (Dekkers et al. 2007). However, the causal relation between the episode of cortisol overexposure and long-term changes in the cardiovascular risk factors is not established and the modifiers of normalization are unknown and are difficult to assess in humans because of the rarity and heterogeneity of CS.

Representative animal models that enable to study the metabolic effects of corticosterone (CORT) overexposure are limited. Whereas in humans, GCs induce weight gain and increase appetite (Chandola et al. 2006), in rats high CORT concentrations (using either chronic stressors or via implantation of subcutaneous [sc] CORT pellets or intraperitoneal [ip] injections) decrease intake of standard chow (Bell et al. 2000). These catabolic effects of GC can be counteracted by adding 30% sucrose to regular chow of CORT-treated rats, and diet (e.g., the addition of sucrose to chow) in fact appears to be a crucial factor for the development of obesity in rats exposed to high CORT. Indeed, when CORT-treated rats with sc pellets were given regular chow with the addition of sucrose, they not only developed visceral obesity but also became markedly hyperinsulinemic and hypercholesterolemic (Bell et al. 2000; Christ-Crain et al. 2008).

Chronic oral CORT supplementation was recently reported to induce impressive metabolic changes in mice including weight gain, increased adiposity, elevated plasma, insulin and triglyceride levels, and hyperphagia (Karatsoreos et al. 2010). As these features resemble the changes observed in individuals suffering from MetS, this represents a model for hypercortisolemia and stress-related obesity.

In the present study, we hypothesized that a period of overexposure to GC in mice would result in long-term or even permanent metabolic changes, and that this would be affected by the composition of the diet. We show that 8 weeks after overexposure to GC, plasma insulin levels remained significantly higher both on low-fat diet (LFD) as well as on high-fat diet (HFD). Furthermore, we show that overexposure to GC persistently altered body composition, but only in the presence of a HFD.

## Materials and Methods

### Animals

Eight-week-old male C57Bl/6J mice (Charles River, Maastricht, The Netherlands) were single housed in a separate room from other experimental animals in the facility to minimize environmental stressors, and maintained on a 12 h:12 h light-dark cycle (lights on 7 a.m.), with ad libitum access to food and drinking water. All animal experiments were performed in accordance with the regulations of Dutch law on animal welfare and the institutional ethics committee for animal procedures from the Leiden University Medical Center approved the protocol.

#### Experiment 1 (LFD experiment)

C57Bl/6J mice (*n* = 20) were fed a LFD (10% kcal% fat, D12451B, Research Diet Services, Inc., New Brunswick, NJ) and after 4 weeks (at 12 weeks of age), they were matched for body weight, the levels of plasma triglycerides, nonesterified free fatty acids, and cholesterol, and randomized to receive either 50 μg/mL CORT (Sigma-Aldrich, Manchester, U.K.) in the drinking water (CORT group, *n* = 10) (containing 0.25% ethanol as vehicle), or 0.25% ethanol as vehicle only in the drinking water (control group, *n* = 10), for 4 weeks. At week 12, dual energy X-ray absorptiometry (DEXA) analysis was performed to determine body composition. Hyperinsulinemic-euglycemic clamp analysis was performed to determine insulin sensitivity (control group *n* = 10, CORT group *n* = 10).

#### Experiment 2 (HFD experiment)

C57Bl/6J mice (*n* = 20) were fed a HFD (45% kcal% fat, D12451, Research Diet Services, Inc., New Brunswick, NJ) from 8 weeks of age and after reaching 12 weeks of age, were matched for weight, plasma triglycerides, nonesterified free fatty acids, and cholesterol, and randomized to receive either 50 μg/mL CORT (Sigma-Aldrich) in the drinking water (CORT group, *n* = 10) (containing 0.25% ethanol as vehicle), or to drinking water with 0.25% ethanol as vehicle only (control group, *n* = 10), for 4 weeks. At week 12, DEXA analysis was performed to determine body composition. Hyperinsulinemic-euglycemic clamp analysis was performed to determine the insulin sensitivity in a subset of mice (control group *n* = 6, CORT group *n* = 6).

### Circadian sampling of plasma levels

Blood for analysis of plasma CORT levels was sampled in both experiments at baseline, and at weeks 4, 8, and 12 during the first light hour at 0700 h, at 1200 h, during the last light hour at 1800 h, and at 2200 h (3 h after the onset of the dark phase). During the dark phase, samples were collected in red light conditions. All CORT samples were obtained within 90 sec from disturbing the cage, via tail incision, allowing the mouse to move freely on top of the home cage (Dalm et al. 2008). Plasma insulin, glucose, total cholesterol, triglycerides, and nonesterified free fatty acids were sampled after overnight fast at baseline, weeks 4, 8, and 12. Body weight, food intake, and water intake were measured weekly.

### DEXA scan

Body composition was measured at week 12 by DEXA using the Norland pDEXA Sabre X-Ray Bone Densitometer (Norland, Hampshire, U.K.). Before measuring, mice were anesthetized with a combination of 6.25 mg/kg acepromazine (Alfasan, Woerden, the Netherlands), 6.25 mg/kg midazolam (Roche, Mijdrecht, the Netherlands), and 0.31 mg/kg fentanyl (Janssen-Cilag, Tilburg, the Netherlands). Mice were scanned in toto, and the heads were subsequently excluded from the analysis due to the inability of the DEXA scan to accurately determine the composition of the tissue underneath the skull.

### Hyperinsulinemic-euglycemic clamp

Hyperinsulinemic-euglycemic clamp studies were performed at week 12 in postabsorptive (i.e., overnight fasted) condition. Mice were anesthetized with 6.25 mg/kg acepromazine (Alfasan, Woerden, the Netherlands), 6.25 mg/kg midazolam (Roche, Mijdrecht, the Netherlands), and 0.31 mg/kg fentanyl (Janssen-Cilag, Tilburg, the Netherlands). First, the basal rate of glucose turnover was determined by giving a primed (0.5 μCi) continuous (0.9 μCi/h) intravenous (iv) infusion of d-[3-^3^H]-glucose (37 MBq) (GE Healthcare, Little Chalfont, U.K.) for 60 min. Subsequently, insulin (Novo Nordisk, Bagsværd, Denmark) was administered in a primed (3.7 mU) continuous 6.1 mU/h for LFD-fed mice and 11.25 mU/h for HFD-fed mice as iv infusion for 90 min to attain steady-state circulating insulin levels. Every 10 min, the plasma glucose concentration was determined via tail vein bleeding (<3 μL) (Accu-chek, Sensor Comfort; Roche Diagnostics GmbH, Mannheim, Germany) and the iv infusion rate of a 12.5% d-glucose solution was adjusted to maintain euglycemia. Blood samples (60 μL) were taken during the basal period (after 50 and 60 min) and during the hyperinsulinemic period (after 70, 80, and 90 min) to determine plasma concentrations of glucose, insulin, and specific activity of ^3^H-glucose. The mice were sacrificed at the end of the clamp.

### Plasma analysis

Plasma CORT levels were determined by radioimmunoassay (MP Biomedicals LCC, Orangeburg, NY). Plasma levels of total cholesterol, triglycerides, and nonesterified free fatty acids were measured with enzymatic colorimetric reaction (Roche diagnostics GmbH, Mannheim; and Wako Pure Chemical Industries, respectively), plasma insulin was measured with an ELISA (Crystal Chem Inc., Downers Grove, IL), and plasma glucose with a hexokinase method (Instruchemie, Delfzijl, The Netherlands). Homeostasis model index of insulin (HOMA-IR) was calculated by multiplying fasting insulin concentration (μU/mL) with fasting glucose (mmol/L), and dividing with 22.5 (Mather 2009).

### Calculations and statistical analysis

Data from the hyperinsulinemic-euglycemic clamp studies were calculated as previously described by Voshol et al. (2001). The rate of glucose uptake (μmol/min per kilogram) was calculated in the basal period as well as during the steady-state hyperinsulinemia (70, 80, and 90 min of the clamp) as the rate of tracer infusion (dpm/min) divided by the plasma-specific activity of ^3^H-glucose (dpm/μmol) in the plasma. Endogenous glucose production (μmol/min per kilogram) was calculated as the difference between the tracer-derived rate of glucose uptake and the glucose infusion rate. Glucose uptake as well as glucose production was corrected for body weight.

Data are presented as means ± SD. Statistical differences were calculated using Mann–Whitney test for nonparametric data, with GraphPad Prism, version 5.01 (La Jolla, CA). *P* < 0.05 was considered statistically significant.

## Results

### Pilot experiment for the determination of optimal CORT dose

Prior to both experiments, we performed a dose-finding study in mice fed a HFD with 12.5 μg/mL (*n* = 2), 25 μg/mL (*n* = 4), and 50 μg/mL (*n* = 2) of CORT and control receiving 0.25% ethanol (*n* = 2) as vehicle for 4 weeks to determine the optimal CORT dose. These dosages were chosen based upon a previous study that documented profound metabolic effects with CORT 100 μg/mL and less pronounced effects with 25 μg/mL (Karatsoreos et al. 2010). Based upon our dose-finding study, we chose 50 μg/mL CORT in the drinking water for our subsequent experiments as this dose led to the most profound increases in food intake and body weight as well as in plasma cholesterol (data not shown).

### CORT treatment increases plasma CORT concentrations and affects circadian rhythm of CORT

**Figure 1:**
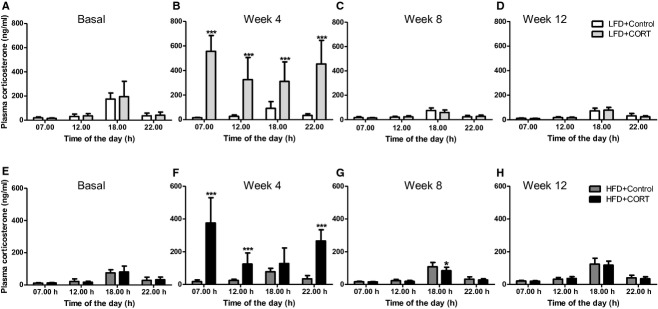
Effect of CORT treatment on circadian plasma CORT of mice fed a low-fat diet (A–D) (control group: open bars, CORT group: light gray bars) or high-fat diet (E–H) (control group: dark gray bars, CORT group: black bars) at baseline, week 4, week 8, and week 12, Mann–Whitney test, *P < 0.05, ***P < 0.001.

A circadian rhythm of CORT was present at baseline in both dietary conditions (Fig. 1A and E) and chronic high doses of CORT (50 μg/mL) increased plasma CORT levels and affected circadian rhythm (Fig. 1B and F, respectively). At week 8 (4 weeks after abrogation of CORT), in the LFD experiment CORT-treated animals had recovered and were not different from the controls, whereas in the HFD experiment, the peak (1800 h) circadian (endogenous) CORT concentrations were −22% lower in CORT-treated animals compared to controls (Fig. 1C and G, respectively). At week 12, circadian CORT levels were equal to the controls (Fig. 1D and H).

### CORT treatment induces transient changes in food intake and body weight

#### LFD experiment

**Figure 2:**
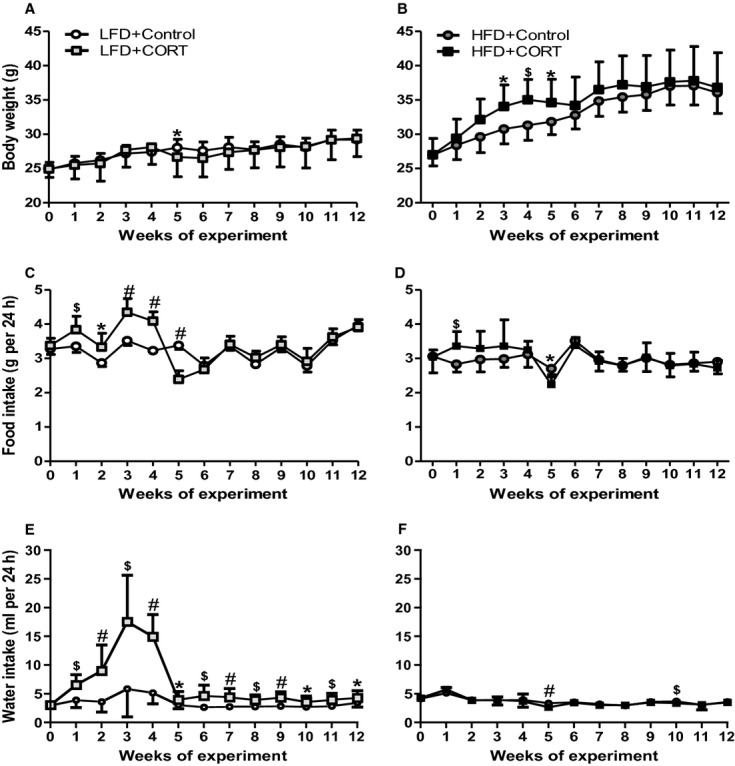
Effect of CORT treatment on body weight, food intake, and water intake of mice fed a low-fat diet (control group: white circles, CORT group: light gray squares) (A, C, and E) or high-fat diet (control group: dark gray circles, CORT group: black squares) (B, D, and F), Mann–Whitney test, *P < 0.05, ^$^*P* < 0.01, ^#^*P* < 0.001.

CORT treatment increased food intake (by 14% vs. controls at week 1 and 27% at week 4) (Fig. 2C). After removal of CORT, food intake rapidly decreased by −29% versus controls returning to the level of the control group at week 6. This increase in food intake was not translated into an increase in body weight, being comparable between the groups during the CORT treatment. Removal of CORT transiently decreased body weight in the CORT group at weeks 5 and 6 (by −5%, and −4%, respectively), but body weight was not different from controls thereafter (Fig. 2A). CORT treatment increased water intake (with CORT in it) up to 3-fold during the treatment period and decreased close to the level of the controls after removal of CORT from the drinking water, however, still remaining significantly higher to the end of the experiment (Fig. 2E).

#### HFD experiment

CORT treatment increased food intake by +19% versus controls after the first week (Fig. 2D), with a concomitant increase in body weight that was significant at week 3 and remained significantly higher at weeks 4 and 5 (+11%, +19%, and +9%, respectively) (Fig. 2B) when compared to the controls. After removal of CORT, food intake normalized at week 6, and did not differ from controls for the remainder of the experiment. CORT treatment did not affect water intake except after removal of CORT from the drinking water at weeks 5 and 10 (by −21% and −10%, respectively) when water intake was significantly lower in the CORT-treated group when compared to controls (Fig. 2F).

### CORT treatment induces transient changes in plasma lipids

CORT treatment increased plasma levels of triglycerides and nonesterified free fatty acids significantly in the LFD experiment at week 4 (by +56% and +24%, respectively) (Table 1). In the HFD experiment, additionally, significant increases versus controls were observed in the plasma levels of total cholesterol and nonesterified free fatty acids at week 4 (+38% and +21%, respectively) (Table 1). At week 8, plasma triglycerides levels were significantly higher in the HFD group (+21% vs. controls) (Table 1).

**Table 1:** Effect of the transient CORT treatment on fasting plasma lipids, insulin, and glucose levels and HOMA-IR of mice fed a low- or high-fat diet

Week	Total cholesterol (mmol/L)				Triglycerides (mmol/L)				Nonesterified free fatty acids (mmol/L)			
		
	LFD		HFD		LFD		HFD		LFD		HFD	
					
	Control	CORT	Control	CORT	Control	CORT	Control	CORT	Control	CORT	Control	CORT
0	3.4 ± 0.3	3.4 ± 0.3	3.2 ± 0.7	3.3 ± 0.4	0.7 ± 0.2	0.7 ± 0.3	1.1 ± 0.4	1.1 ± 0.3	1.4 ± 0.3	1.4 ± 0.3	1.6 ± 0.3	1.8 ± 0.3
4	3.0 ± 0.3	3.2 ± 0.6	3.5 ± 0.7	4.8 ± 0.6^b^	0.9 ± 0.2	1.4 ± 0.3^c^	1.1 ± 0.3	1.3 ± 0.2	1.7 ± 0.3	2.1 ± 0.4^a^	1.8 ± 0.2	2.2 ± 0.3^b^
8	3.1 ± 0.3	2.9 ± 0.4	3.9 ± 0.8	4.0 ± 0.4	0.7 ± 0.1	0.8 ± 0.3	1.5 ± 0.6	1.8 ± 0.4^a^	1.6 ± 0.2	1.5 ± 0.2	1.7 ± 0.3	1.9 ± 0.3
12	2.8 ± 0.6	2.9 ± 0.7	4.1 ± 0.8	4.2 ± 0.9	0.7 ± 0.2	0.8 ± 0.2	1.2 ± 0.2	1.4 ± 0.3	1.4 ± 0.2	1.5 ± 0.3	1.5 ± 0.3	1.7 ± 0.3

	Insulin (ng/mL)				Glucose (mmol/L)				HOMA-IR			
0	0.6 ± 0.3	0.6 ± 0.2	0.6 ± 0.3	0.5 ± 0.2	3.6 ± 0.7	3.8 ± 1.0	5.3 ± 1.1	5.2 ± 0.7	2.3 ± 0.8	2.2 ± 0.9	3.1 ± 1.5	2.7 ± 1.0
4	0.6 ± 0.2	4.8 ± 2.2^c^	1.1 ± 0.3	4.0 ± 1.1^c^	4.1 ± 0.9	3.4 ± 0.7	4.3 ± 0.7	3.4 ± 0.7^a^	2.3 ± 0.6	17.7 ± 10.6^b^	5.0 ± 1.8	14.4 ± 3.7^c^
8	0.6 ± 0.2	1.1 ± 0.8^a^	0.6 ± 0.3	1.0 ± 0.4^a^	4.2 ± 0.6	3.6 ± 0.6^a^	5.2 ± 1.1	5.2 ± 1.2	2.5 ± 0.7	4.3 ± 2.7	3.4 ± 1.6	5.7 ± 2.5^a^
12	0.6 ± 0.4	1.0 ± 0.4^a^	1.3 ± 0.4	2.2 ± 1.0^a^	4.8 ± 0.6	4.0 ± 0.6^b^	3.9 ± 1.1	3.1 ± 0.7	2.9 ± 1.9	4.2 ± 1.7	5.3 ± 2.4	7.4 ± 4.0

Mann–Whitney test,

a *P* < 0.05,

b *P* < 0.01,

c *P* < 0.001.

### Insulin and HOMA-IR are increased long term by CORT treatment

As expected, when compared to controls, CORT treatment significantly increased plasma insulin concentrations on both diets (LFD: 8-fold and HFD: 3-fold increase at week 4) (Table 1), and remained significantly higher after removal of CORT treatment. Of note, insulin levels did not markedly decrease in CORT-treated animals between week 8 (LFD: +83% and HFD: +67%) and week 12 (LFD: +67% and HFD: +69%) (Table 1). Plasma glucose was significantly decreased in the LFD experiment at weeks 8 and 12 (−14% and −17%, respectively) and by −21% in the HFD experiment at week 4 in the CORT groups (Table 1). Changes in plasma insulin and glucose induced by CORT treatment, suggested decreased insulin sensitivity as reflected in HOMA-IR. In both experiments, CORT significantly increased HOMA-IR at week 4 (LFD: 8-fold and HFD: 3-fold) (Table 1). Furthermore, HOMA-IR at week 8 was still significantly increased by +69% in the HFD experiment after CORT treatment (Table 1), and by +45% in the LFD experiment at week 12, which, however, did not reach significance, (Table 1) when compared to the controls (Table 1).

### CORT treatment induces long-lasting changes in body composition only in the presence of HFD

**Figure 3:**
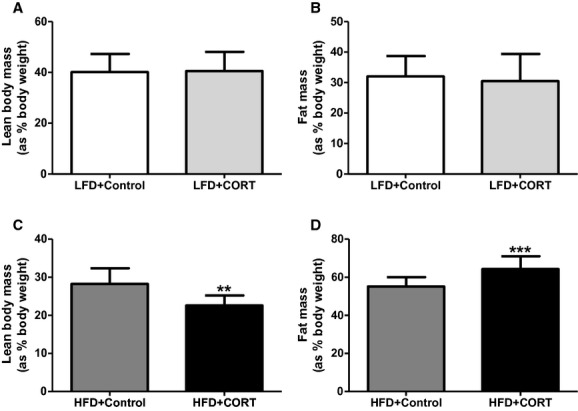
Effect of CORT treatment on body composition as measured by DEXA at week 12 of mice fed a low-fat diet (A and B) (control group: open bars, CORT group: light gray bars) or high-fat diet (C and D) (control group: dark gray bars, CORT group: black bars), Mann–Whitney test, **P < 0.01, ***P < 0.001.

In the LFD experiment, no long-lasting changes in body composition were observed (Fig. 3A and B). In the HFD experiment, after 12 weeks, CORT treatment significantly reduced lean body mass (23 ± 3% vs. 28 ± 4% of total body weight, CORT vs. controls, respectively) (Fig. 3C), and significantly increased fat mass (55 ± 5% vs. 64 ± 7% of the total body weight) (Fig. 3D).

### CORT treatment does not affect endogenous glucose production or glucose disposal in the long term

**Figure 4:**
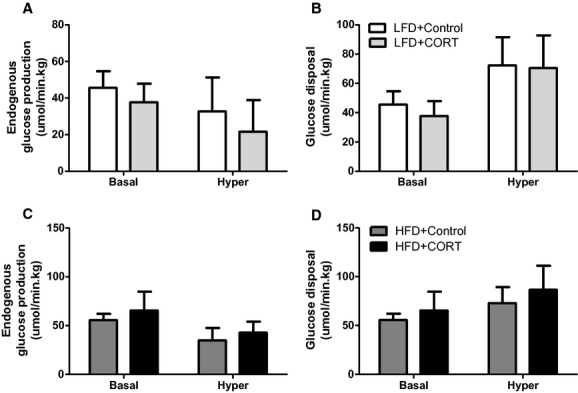
Effect of CORT treatment on endogenous glucose production and glucose disposal during basal period of glucose infusion (basal) and hyperinsulinemia (hyper) in mice fed a low-fat diet (A and B) (control group: open bars, CORT group: light gray bars) or high-fat diet (C and D) (control group: dark gray bars, CORT group: black bars) at week 12.

Endogenous glucose production and glucose disposal, as derived from the hyperinsulinemic-euglycemic clamp studies, were not different between the CORT-treated and the control group in the LFD experiment (Fig. 4A and B), nor in the HFD experiment (Fig. 4C and D) at week 12.

## Discussion

We demonstrated that a period of overexposure to GCs induces long-lasting, potentially permanent, changes in metabolic parameters, but only in the presence of a HFD. In HFD-fed animals, increased insulin levels and altered body composition did not normalize even after an 8-week washout period, when parameters like food intake and body weight had returned to control levels for about 6 weeks.

As anticipated, in our study, high CORT levels increased food intake and induced metabolic changes that resemble the MetS, like an increase in plasma insulin levels, lipids, and HOMA index, irrespective of a HFD or LFD. While some effects were reversible after normalization of CORT levels, insulin was irreversibly increased under both LFD and HFD conditions. Mice on HFD showed additional long-lasting effects of high CORT exposure, that is, adversely affected body composition (even if body weight quickly normalized after cessation of CORT exposure). This indicates that HFD aggravates the adverse long-term metabolic effects of transient high CORT exposure. This points to a crucial role for dietary composition in the development of the MetS in conditions with periodic excessive CORT exposure.

Supraphysiological CORT levels were readily induced using oral CORT in the drinking water. This treatment with CORT also affected circadian CORT rhythm, impairing the degree of variations in plasma concentrations. After discontinuation of CORT treatment, circadian rhythm gradually returned to the level of the controls at week 12, although evening (1800 h) CORT peak was still decreased at week 8 in the HFD experiment. This indicates that incomplete recovery of the HPA axis after a period of exogenous supraphysiological CORT supplementation, as has been documented extensively in humans both after treatment for CS (Pereira et al. 2003) and in patients previously exposed to exogenous supraphysiological GCs (Lamberts et al. 1997), might be dependent on the composition of the diet.

CORT treatment increased food intake on both diets, but body weight increased only on HFD. It has been previously shown that high CORT levels stimulate voluntary food intake dose dependently (Bell et al. 2000; Bhatnagar et al. 2000; la Fleur et al. 2004). In addition, in case of different food availability, food preference changes toward more energy-rich “palatable” food (Pecoraro et al. 2004) serving the evolutionary purpose to attain the most efficient fuels to counteract the adverse changes in insulin sensitivity that occur during stress (Dallman et al. 2003). With increasing insulin and GC levels, food intake is even stimulated in the presence of less palatable foods (Akana et al. 1985; Strack et al. 1995; Bell et al. 2000). In agreement, in the present experiments with LFD and HFD, the increased plasma CORT levels were accompanied by increases in circulating insulin, triglycerides, cholesterol, and free fatty acid levels. The increased food intake resulted in increased body weight only on HFD, suggesting that the increase in food intake on LFD was just sufficient to compensate for the catabolic effects of increased CORT levels. However, higher dosages of CORT than the one used in our study were able to increase body weight on regular chow diet (Karatsoreos et al. 2010). Intriguingly, the increase in fat mass remains present even after a washout from the increased plasma CORT levels.

Water intake and therefore CORT intake were increased only in the LFD but not in the HFD experiment in the CORT-treated group. However, even when consuming a higher amount of CORT during LFD, the reversibility of the metabolic changes after LFD (but not after HFD) was complete, which strengthens our conclusions on the modulatory role of the diets per se.

Although body weight returned to that of the controls after discontinuation of CORT treatment, on HFD the increase in plasma triglycerides was still present at week 8, and insulin concentrations remained elevated relative to controls in both diet groups even at week 12. However, endogenous glucose production and disposal assessed with hyperinsulinemic-euglycemic clamps at the end of the study did not indicate reduced insulin sensitivity.

Whereas the effects on body weight were transient, long-lasting changes in body composition were found on HFD, where reduced lean body mass and increased fat mass were observed even after 12 experimental weeks. These effects of CORT treatment on body composition were not observed in the LFD experiment. Chronically administered GCs facilitate muscle atrophy (Watson et al. 2012) and increase visceral fat mass in mice (Gounarides et al. 2008; Karatsoreos et al. 2010). In the human, the equivalent of chronic high GC is CS, a rare clinical syndrome where patients are exposed to increased adrenal cortisol secretion due to an ACTH-producing pituitary or ectopic tumor or due to an adrenal adenoma (Newell-Price et al. 2006). Patients with CS have increased prevalence of the MetS (Arnaldi et al. 2003; van Raalte et al. 2009), but intriguingly, after 1 year of remission still have increased waist circumference (Faggiano et al. 2003; Giordano et al. 2011) and even after long-term remission higher visceral fat mass was observed without affecting body mass index (Barahona et al. 2009).

The fact that the long-lasting changes observed in mice after a period of exposure to high CORT persist for a longer period of time in the presence of HFD is intriguing in view of the incomplete reversibility of metabolic changes observed in patients with CS after correction for hypercortisolism. Humans exposed to stress levels of GC, like in CS, will direct their food preference toward highly palatable foods, in agreement with the biological effects of GC, thereby aggravating the adverse cardiometabolic effects and thus facilitating the development and persistence of MetS. Epidemiological data also indicate an increased prevalence of the MetS in conditions associated with alterations in the HPA axis, like in anxiety and depression, after medical treatment with GC, and also in sleep disorders (Balbo et al. 2010; Sahar and Sassone-Corsi 2012). As it appears that these diet-dependent effects of CORT during stress strongly facilitate the persistent adverse cardiovascular risk profile, the interactions between the availability of “fast food” and every-day-stress are novel features to be accentuated in future studies on cardiovascular morbidity and mortality.

## References

[b1] Akana SF, Cascio CS, Shinsako J, Dallman MF (1985). CORT: narrow range required for normal body and thymus weight and ACTH. Am. J. Physiol.

[b2] Arnaldi G, Angeli A, Atkinson AB, Bertagna X, Cavagnini F, Chrousos GP (2003). Diagnosis and complications of Cushing's syndrome: a consensus statement. J. Clin. Endocrinol. Metab.

[b3] Balbo M, Leproult R, Van Cauter E (2010). Impact of sleep and its disturbances on hypothalamo-pituitary-adrenal axis activity. Int. J Endocrinol.

[b4] Barahona MJ, Sucunza N, Resmini E, Fernández-Real JM, Ricart W, Moreno-Navarrete JM (2009). Persistent body fat mass and inflammatory marker increases after long-term cure of Cushing's syndrome. J. Clin. Endocrinol. Metab.

[b5] Bell ME, Bhatnagar S, Liang J, Soriano L, Nagy TR, Dallman MF (2000). Voluntary sucrose ingestion, like CORT replacement, prevents the metabolic deficits of adrenalectomy. J. Neuroendocrinol.

[b6] Bhatnagar S, Bell ME, Soriano L, Nagy TR, Dallman MF (2000). CORT facilitates saccharin intake in adrenalectomized rats. Does CORT increase stimulus salience?. J. Neuroendocrinol.

[b7] Chandola T, Brunner E, Marmot M (2006). Chronic stress at work and the metabolic syndrome: prospective study. BMJ.

[b8] Christ-Crain M, Kola B, Lolli F, Fekete C, Seboek D, Wittmann G (2008). AMP-activated protein kinase mediates glucocorticoid-induced metabolic changes: a novel mechanism in Cushing's syndrome. FASEB J.

[b9] Dallman MF, Pecoraro N, Akana SF, Gomez SE, La Fleur F, Houshyar H (2003). Chronic stress and obesity: a new view of “comfort food”. Proc. Natl. Acad. Sci. USA.

[b10] Dalm S, Brinks V, Oitzl MH, van der Mark ER, de Kloet MS (2008). Non-invasive stress-free application of glucocorticoid ligands in mice. J. Neurosci. Methods.

[b11] Dekkers OM, Biermasz NR, Pereira AM, Roelfsema F, Voormolen MO, van Aken JH (2007). Mortality in patients treated for Cushing's disease is increased, compared with patients treated for nonfunctioning pituitary macroadenoma. J. Clin. Endocrinol. Metab.

[b12] Faggiano A, Pivonello R, Spiezia S, Filippella MC, De Martino M, Di Somma C (2003). Cardiovascular risk factors and common carotid artery caliber and stiffness in patients with Cushing's disease during active disease and 1 year after disease remission. J. Clin. Endocrinol. Metab.

[b13] la Fleur SE, Akana SF, Manalo S, Dallman MF (2004). Interaction between CORT and insulin in obesity: regulation of lard intake and fat stores. Endocrinology.

[b14] Giordano R, Picu A, Marinazzo E, D'Angelo V, Berardelli R, Karamouzis I (2011). Metabolic and cardiovascular outcomes in patients with Cushing's syndrome of different aetiologies during active disease and 1 year after remission. Clin. Endocrinol. (Oxf).

[b15] Gounarides JS, Korach-André M, Killary K, Argentieri G, Turner O, Laurent D (2008). Effect of dexamethasone on glucose tolerance and fat metabolism in a diet-induced obesity mouse model. Endocrinology.

[b16] Karatsoreos IN, Bhagat SM, Bowles NP, Weil ZM, Pfaff DW, McEwen BS (2010). Endocrine and physiological changes in response to chronic CORT: a potential model of the metabolic syndrome in mouse. Endocrinology.

[b17] Lamberts SW, Bruining HA, de Jong FH (1997). Corticosteroid therapy in severe illness. N. Engl. J. Med.

[b18] Mather K (2009). Surrogate measures of insulin resistance: of rats, mice, and men. J. Clin. Endocrinol. Metab.

[b19] Newell-Price J, Bertagna X, Grossman AB, Nieman LK (2006). Cushing's syndrome. Lancet.

[b20] Pecoraro N, Reyes F, Gomez F, Bhargava A, Dallman MF (2004). Chronic stress promotes palatable feeding, which reduces signs of stress: feedforward and feedback effects of chronic stress. Endocrinology.

[b21] Pereira AM, Schutte MO, van Aken H, van Dulken PJ, Biermasz NR, Smit JW (2003). Long-term predictive value of postsurgical cortisol concentrations for cure and risk of recurrence in Cushing's disease. J. Clin. Endocrinol. Metab.

[b22] van Raalte DH, Ouwens DM, Diamant M (2009). Novel insights into glucocorticoid-mediated diabetogenic effects: towards expansion of therapeutic options?. Eur. J. Clin. Invest.

[b23] Sahar S, Sassone-Corsi P (2012). Regulation of metabolism: the circadian clock dictates the time. Trends Endocrinol. Metab.

[b24] Strack AM, Sebastian RJ, Schwartz MW, Dallman MF (1995). Glucocorticoids and insulin: reciprocal signals for energy balance. Am. J. Physiol.

[b25] Voshol PJ, Jong MC, Dahlmans VE, Kratky D, Levak-Frank S, Zechner R (2001). In muscle-specific lipoprotein lipase-overexpressing mice, muscle triglyceride content is increased without inhibition of insulin-stimulated whole-body and muscle-specific glucose uptake. Diabetes.

[b26] Warne JP, Akana SF, Ginsberg AB, Horneman HF, Pecoraro NC, Dallman MF (2009). Disengaging insulin from CORT: roles of each on energy intake and disposition. Am. J. Physiol. Regul. Integr. Comp. Physiol.

[b27] Watson ML, Baehr LM, Reichardt HM, Tuckermann JP, Bodine SC, Furlow JDA (2012). Cell autonomous role for the glucocorticoid receptor in skeletal muscle atrophy induced by systemic glucocorticoid exposure. Am. J. Physiol. Endocrinol. Metab.

